# Long-Term Investigation into the Membrane Fouling Behavior in Anaerobic Membrane Bioreactors for Municipal Wastewater Treatment Operated at Two Different Temperatures

**DOI:** 10.3390/membranes10090231

**Published:** 2020-09-13

**Authors:** Yi Ding, Zhansheng Guo, Zhenlin Liang, Xuguang Hou, Zhipeng Li, Dashuai Mu, Changzi Ge, Chunpeng Zhang, Chao Jin

**Affiliations:** 1 Marine College, Shandong University, Weihai 264209, China; dingyi@sdu.edu.cn (Y.D.); guozhansheng@sdu.edu.cn (Z.G.); liangzhenlin@sdu.edu.cn (Z.L.); richardhoukk@163.com (X.H.); dashuai.mu@sdu.edu.cn (D.M.); changzige@sdu.edu.cn (C.G.); 2 State Key Laboratory of Urban Water Resources and Water Environment, School of Marine Science and Technology, Harbin Institute of Technology at Weihai, Weihai 264200, China; 3 Key Laboratory of Groundwater Resources and Environment (Ministry of Education), Jilin University, Changchun 130021, China; zhang_cp@jlu.edu.cn; 4 School of Environmental Science and Engineering, Sun Yat-Sen University, Guangzhou 510275, China

**Keywords:** anaerobic membrane bioreactor, temperature, membrane fouling behavior, sludge flocs characteristics, soluble microbial product, extracellular polymeric substance

## Abstract

In this study, the characteristics of activated sludge flocs were investigated and their effects on the evolution of membrane fouling were considered in the anaerobic membrane bioreactors (AnMBR), which were operated at 25 and 35 °C for municipal wastewater treatment. It was found that the membrane fouling rate of the AnMBR at 25 °C was more severe than that at 35 °C. The membrane fouling trends were not consistent with the change in the concentration of soluble microbial product (SMP). The larger amount of SMP in the AnMBR at 35 °C did not induce more severe membrane fouling than that in the AnMBR at 25 °C. However, the polysaccharide and protein concentration of extracellular polymeric substance (EPS) was higher in the AnMBR at 25 °C in comparison with that in the AnMBR at 35 °C, and the protein/polysaccharide ratio of the EPS in the AnMBR at 25 °C was higher in contrast to that in the AnMBR at 35 °C. Meanwhile, the fouling tendencies measured for the AnMBRs could be related to the characteristics of loosely bound EPS and tightly bound EPS. The analysis of the activated sludge flocs characteristics indicated that a smaller sludge particle size and more fine flocs were observed at the AnMBR with 25 °C. Therefore, the membrane fouling potential in the AnMBR could be explained by the characteristics of activated sludge flocs.

## 1. Introduction

Membrane separation technology coupled with an anaerobic bioreactor can be used for municipal sewage treatment [1]. The anaerobic membrane bioreactor (AnMBR) has attracted a lot of attention due to the advantages of less sludge production, higher loading rate, better quality effluent, and lower energy consumption [2]. The anaerobic process can be operated under different temperature [3]. As a result of the slow anaerobic microorganism growth, a long sludge retention time (SRT) is needed to achieve better pollutant removal efficiency, especially for municipal wastewater treatment [4]. Although the SRT should be longer than that commonly used at mesophilic temperatures, AnMBR operation near room temperature is technically feasible for municipal wastewater treatment [5].

However, due to the limitation of anaerobic microbial metabolism at 25 °C, the increased colloid and dissolved solids content during the anaerobic process may enhance the membrane fouling propensity. Membrane fouling still is one of the key problems of membrane bioreactor research. Extracellular polymeric substances (EPS) and soluble microbial products (SMP) are the major causes of membrane fouling. SMPs are the soluble cellular components secreted by microorganisms, and the EPSs have been differentiated into the inner layer and the outer layer [6]. The inner layer is mainly composed of tightly bound EPSs (TB-EPS), and the outer layer is mainly referred to as loosely bound EPSs (LB-EPS) [7]. The content of the LB-EPS and TB-EPS has some effect on the microbial aggregates [8]. Moreover, some authors indicated that the protein and carbohydrate were considered to be the main reason for flux reduction [9,10], and other researchers observed that pore blockage and cake layer formation were significantly enhanced by EPS during MBR operation [11,12]. It has been reported that the proteinous and carbohydrate EPSs and SMPs were strongly correlated with the type of wastewater [13,14].

Therefore, the purpose of this research was to discuss the membrane fouling mechanisms in an anaerobic membrane bioreactor for municipal wastewater treatment under two different temperature conditions. We sought to (1) assess the membrane fouling behavior; (2) investigate the EPS and SMP characteristics, and (3) analyze the size distribution and morphology of the sludge flocs. This study would further improve the understanding of membrane fouling behaviors in AnMBR for wastewater treatment.

## 2. Materials and Methods

### 2.1. AnMBRs Operating at Two Different Temperatures

The anaerobic process can be conducted at psychrophilic, mesophilic, and thermophilic temperature ranges [15]. Under the mesophilic condition, the reactor was usually operated at 35 °C [16]. During the conventional experiment, the MBR was often operated under the room temperature condition, at a temperature of 25 ± 0.5 °C [17]. Therefore, two identical AnMBRs operated at 25 and 35 °C were used in this study to discuss the membrane fouling mechanisms in an anaerobic membrane bioreactor for municipal wastewater treatment under different temperature conditions. The experimental set-up of the AnMBRs at 25 and 35 °C is shown in a previous study [18]. The cylindrical anaerobic MBR was made of a polymethyl methacrylate, and the volume was 8.0 L. The AnMBR was equipped with a rounding polyvinylidene fluoride (PVDF) membrane module with a membrane pore size of 0.22 μm and membrane surface area of 0.2 m^2^. A water level controller was utilized to maintain the wastewater volume. The transmembrane pressure (TMP) was recorded by a vacuum meter (YB150, Yangquan, China). The TMP data presented were based on the measurements conducted after the AnMBRs reached steady state. The steady state herein refers to the experimental period approximately after 200 days. Once the TMP reached 30 kPa in the AnMBRs, the membrane modules were taken out and cleaned. The modules were reloaded into the bioreactors to run the next hydraulic retention time (HRT) after cleaning. Furthermore, the effluent pump was operated intermittently in scheduled mode. The bioreactor temperature was maintained at scheduled temperatures.

### 2.2. Operating Parameters of the AnMBRs

Simulated municipal sewage was used as feed water for the AnMBRs at 25 and 35 °C, according to previous study [18]. Activated sludge from sewage treatment plant (Harbin, China) was used as inoculum for the AnMBRs at 25 and 35 °C. The sludge retention time (SRT) and the HRT were maintained at 370 days and 24 h, respectively. The sludge concentration (MLSS) was 5861 mg/L and 6024 mg/L for the AnMBR at 25 and 35 °C, respectively, and the MLSS concentrations of the AnMBRs had little change during the whole long-term operation process.

### 2.3. SMP and EPS Preparation from Anaerobic Membrane Bioreactor

The SMP and EPS was prepared based on the following procedure. First, the sludge mixture was centrifuged for 5 min with 5000 rpm. Second, the collected supernatant was filtered by microporous membrane. The collected filtrate was considered to be SMP. The LB-EPS and TB-EPS were extracted according to previous research and measured for the amount of proteins and carbohydrates [19]. EPS content was characterized by the sum of protein and polysaccharide per gram of dry sludge. All the above analyses were performed in triplicate, and their average values were listed.

### 2.4. Analytical Methods

Proteins and carbohydrates were analyzed by the Lowry method [20] and the phenol–sulfuric method [21], respectively. Excitation–emission matrix (EEM) spectra (FP 6500, JASCO, Tokyo, Japan) were obtained according to a previous study [22]. The morphological characteristics of the activated sludge were investigated by the floc size distribution and sludge flocs morphology. Particle size distribution (PSD) was analyzed through a Mastersizer 2000 coupled to Hydro 2000SM (A) with a detection range of 0.02–2000 μm (Mastersizer 2000, Malverin, England). The sludge floc morphology was investigated by microscopy (BX51, Olympus, Tokyo, Japan) and the images were obtained. The EEM spectra and PSD were conducted in triplicate, and only the representative results are reported in the paper. In total, 12 different sludge floc morphology images of each sample were obtained, and the representative images are shown in the research.

## 3. Results and Discussion

### 3.1. Membrane Fouling Behavior

**Figure 1:**
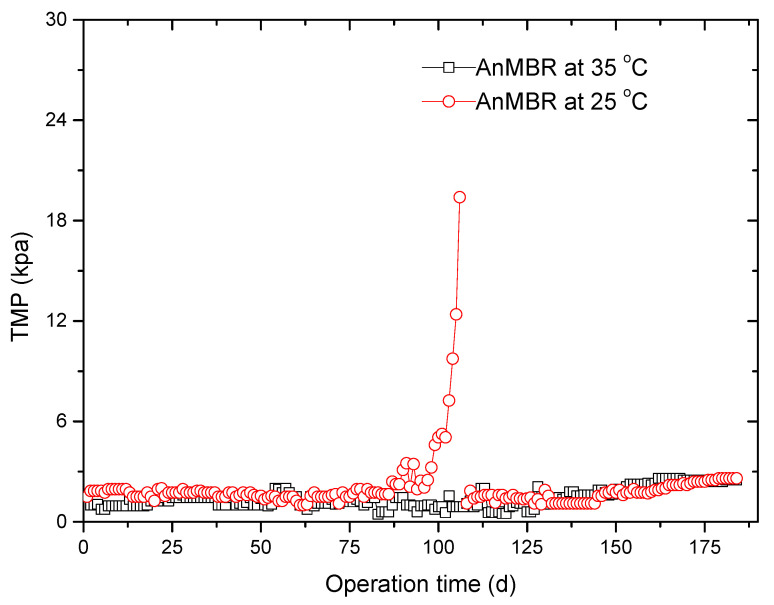
The variations in transmembrane pressure (TMP) throughout the experimental period.

The changes in TMP throughout the experimental period are illustrated in Figure 1. The TMP generally increased with time and reached low values (1.45 kpa for the AnMBR at 35 °C, 3.1 kpa for the AnMBR at 25 °C) at the initial 90 d in the two AnMBRs. However, the TMP jumped to 19.4 kpa at 106 d in the AnMBR at 25 °C, while the TMP in the AnMBR at 35 °C remained stable and never underwent transition during the 180-day operation. Obviously, the membrane fouling rate of the AnMBR at 25 °C increased more slowly than that of the AnMBR at 35 °C. The mixed liquor in the AnMBR at 25 °C exhibited consistently higher membrane fouling propensity than the mixed liquor in the AnMBR at 35 °C. It is believed that membrane fouling is mainly induced by SMP and EPS [23,24]. We aimed to clarify the reason for different membrane fouling rates between the AnMBR at 25 °C and the AnMBR at 35 °C; the characteristics of the SMP and EPS are studied in the following sections.

### 3.2. Changes in Concentrations of SMP

**Figure 2:**
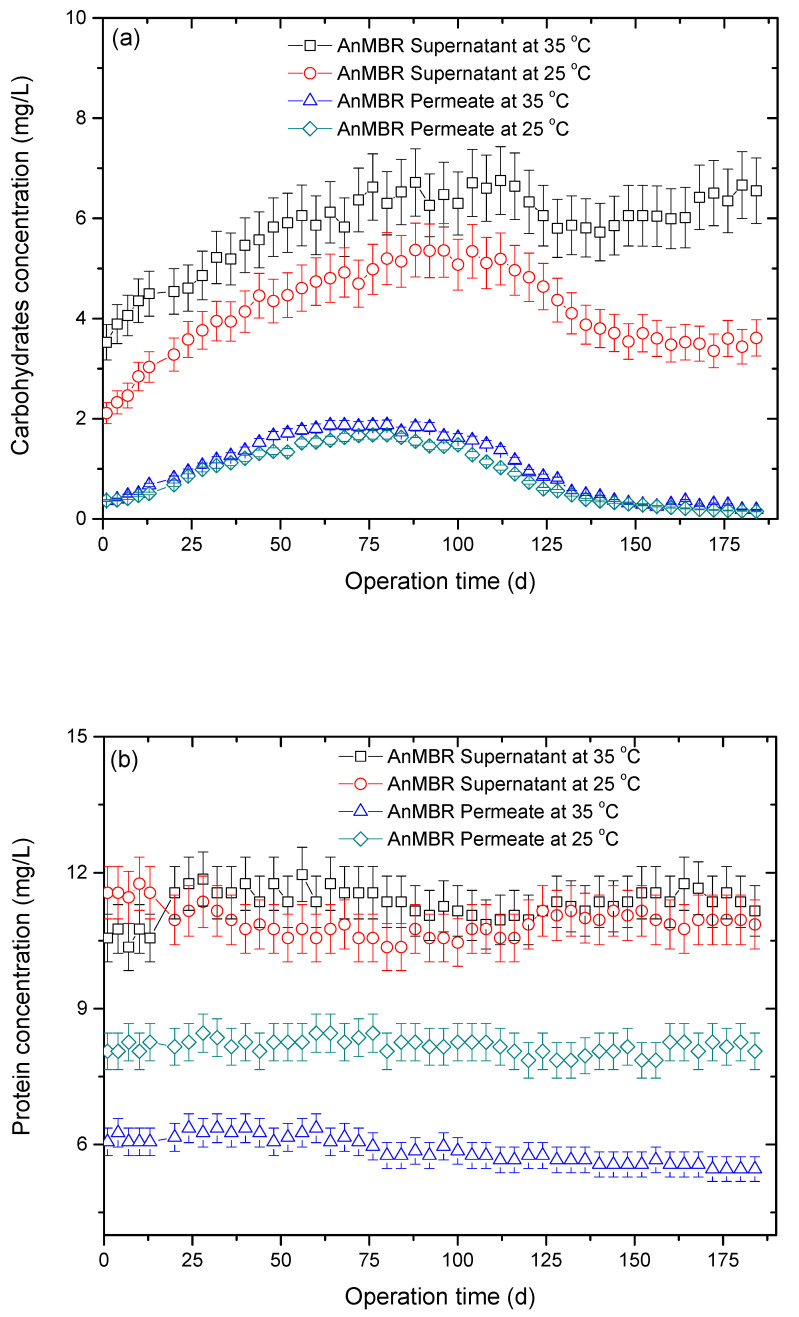
The changes in carbohydrate (**a**) and protein (**b**) contents in soluble microbial products (SMP) during the operation time.

The variations in carbohydrate and protein contents for SMP are illustrated in Figure 2 during the operation time. This shows that the content of SMP of the two AnMBRs at 35 and 25 °C had the same tendency. Figure 2a illustrates the variations in the carbohydrate concentration in SMP throughout the experimental period. It was found that the carbohydrate concentrations were increased in the beginning 120 days in the supernatant and permeate of the anaerobic membranes, and then the carbohydrate concentrations were kept relatively stable in the following period.

Figure 2b shows the protein concentration in SMP of the two AnMBRs at 35 and 25 °C. The protein content seemed to be less affected by the long-term operation. Though the protein content in SMP of the AnMBR at 35 °C was slightly higher than that in SMP of the AnMBR at 25 °C, the protein content in the permeate of the AnMBR at 35 °C was dramatically lower than that in the permeate of the AnMBR at 25 °C. Although the carbohydrate and protein contents of the AnMBR at 35 °C in the supernatant were slightly higher than those of the AnMBR at 25 °C, this difference was small and was assumed not to cause a significant change in sludge filterability. Therefore, the membrane fouling trends were not consistent with the change in the concentrations of SMP, and the content of SMP was obviously not indicative of the fouling tendencies of the two AnMBRs.

### 3.3. Changes in Concentrations of EPS

**Figure 3:**
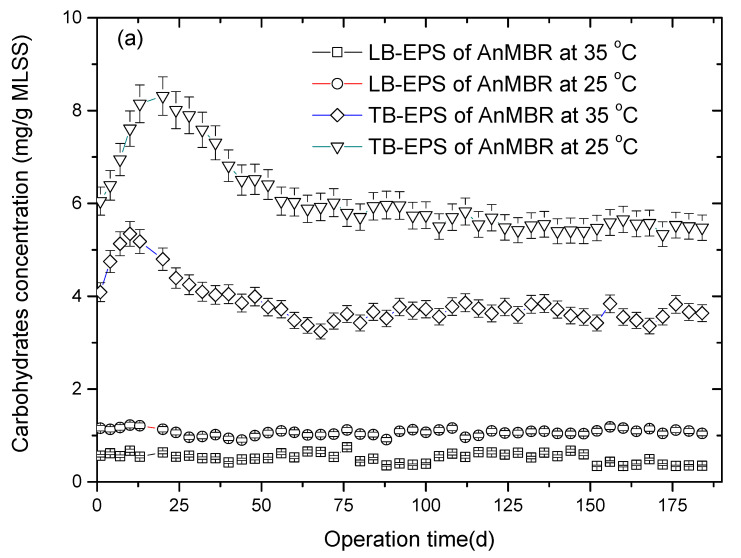

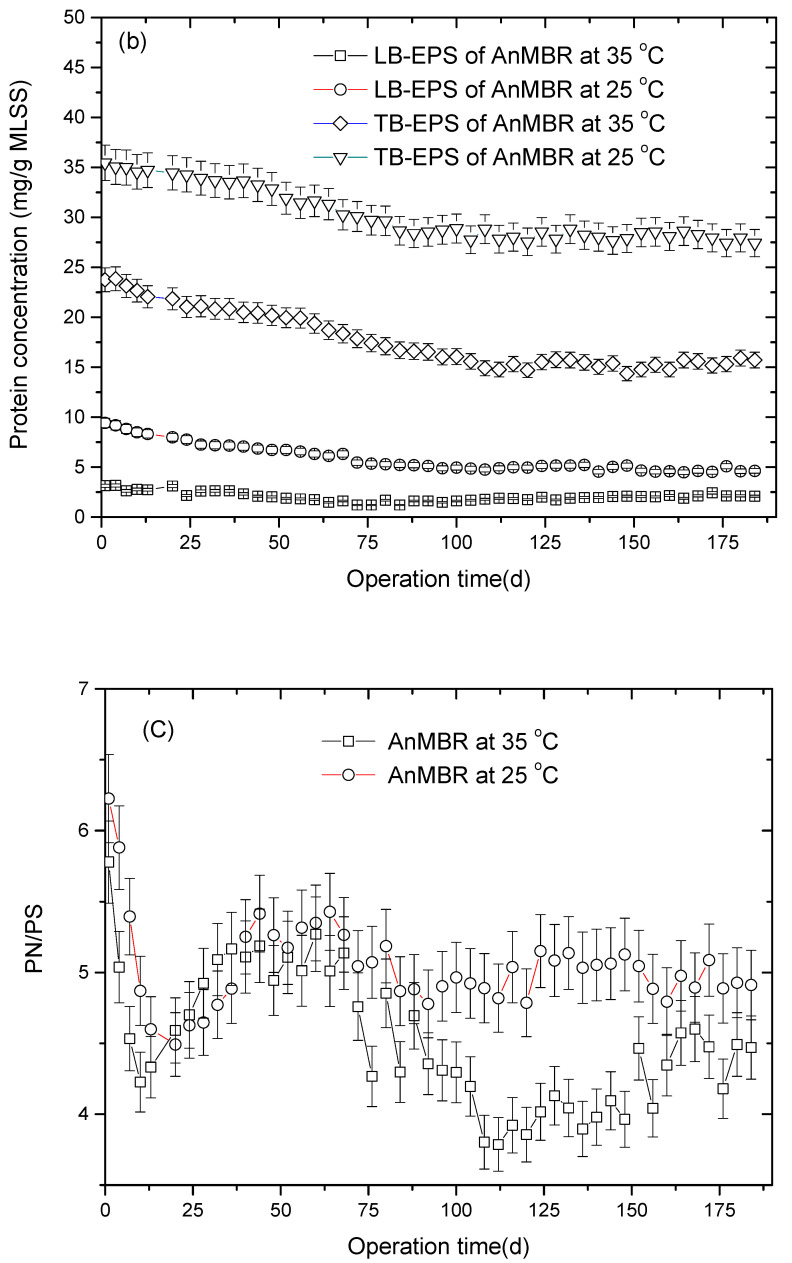
The variations in carbohydrate (**a**) and protein (**b**) contents and the proteins (PN)/polysaccharides (PS) ratio for extracellular polymeric substances (EPS) (**c**) in the two anaerobic membrane bioreactors (AnMBRs) at 35 and 25 °C against the operation time.

EPS was commonly considered to be the main reason for membrane fouling in MBR [25]. The content changes in carbohydrate and protein of LB-EPS and TB-EPS are shown in Figure 3a,b in the two AnMBRs at 35 and 25 °C against the operation time. It can be seen from Figure 3a that the carbohydrate in TB-EPS was more than that in LB-EPS in both the two AnMBRs at 35 and 25 °C. Meanwhile, the carbohydrate in LB-EPS and TB-EPS of the AnMBR at 35 °C was less than that in LB-EPS and TB-EPS of the AnMBR at 25 °C.

The protein content of LB-EPS and TB-EPS in the two AnMBRs at 35 and 25 °C is listed in Figure 3b. It was found that the protein concentrations declined slowly for the LB-EPS and TB-EPS in the two AnMBRs at 35 and 25 °C, but the values for the LB-EPS and TB-EPS in the AnMBR at 25 °C were significantly higher than those in the AnMBR at 35 °C. It was seen that protein was the main component of the EPS. It has been reported that LB-EPS plays a more important role in membrane fouling compared to TB-EPS [26]. In this research, the content of LB-EPS and TB-EPS in the sludge flocs showed a relationship with the fouling tendency. The polysaccharide and protein concentration of LB-EPS and TB-EPS were higher at the AnMBR with 25 °C compared those at AnMBR with 35 °C, which may result in the faster fouling propensity in AnMBR at 25 °C.

The variation in the proteins (PN)/polysaccharides (PS) ratio for EPS with operation time is presented in Figure 3c. Obviously, the PN/PS ratio for EPS of AnMBR at 35 °C was lower than that for EPS of AnMBR at 25 °C. In the course of the experiment, the average PN/PS ratio for EPS in the AnMBR at 35 °C was 4.88, which was 10% lower than that in the AnMBR at 25 °C (5.40). It has been found that the PN/PS ratio of EPS could indicate the membrane fouling trend of sludge flocs [27]. Therefore, with respect to the AnMBR at 35 °C, the lower PN/PS ratio for the EPS could lead to less membrane fouling than that in the AnMBR at 25 °C. Additionally, it had been reported that the PN/PS ratio reduction of EPS could cause a decrease in floc hydrophobicity [27]. It was for this reason that the sludge deposition on the membranes was reduced and the fouling layer caused by the sludge flocs would be mitigated in the AnMBR at 35 °C.

In the present study, mixed liquor samples with higher polysaccharide and protein content and protein/polysaccharide ratio in EPS were observed to have greater potential to foul membranes during the AnMBR operation at 25 °C. It was suggested that the EPS in the mixed liquor, particularly the carbohydrates and protein substances, were the major substances that accumulated on the membranes and consequently caused membrane fouling.

### 3.4. Characteristics of SMP and EPS

**Figure 4:**
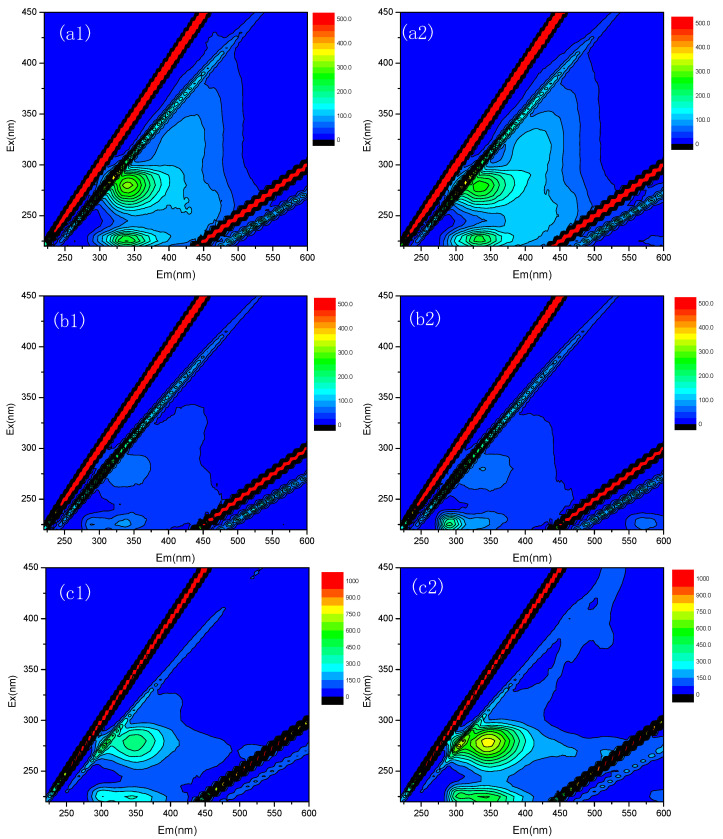
Excitation–emission matrix (EEM) spectra of SMP (**a**), loosely bound EPS (LB-EPS) (**b**) and tightly bound EPS (TB-EPS) (**c**) in the AnMBR at 35 °C (1) and the AnMBR at 25 °C (2).

EEM analysis was used to study the characteristics of SMP and EPS in the two AnMBRs at 35 and 25 °C, which provides spectral information about the aromatic protein-like and tryptophan protein-like compounds [28]. It can be seen from Figure 4 that the EEM spectra all showed two peaks for the SMP and EPS in the mixed liquor suspension of the two AnMBRs at 35 and 25 °C. At the excitation/emission wavelengths (Ex/Em) of 230–240 nm/335–350 nm (Peak A), the peak was considered to reflect aromatic protein-like substances, and at the Ex/Em of 280 nm/330–340 nm (Peak B), the peak was associated with the tryptophan protein-like substances [29].

It was found that the peak relative dominance for protein-like substances in the SMP extracted from AnMBR at 35 °C was stronger than that extracted from AnMBR at 25 °C; however, the peak intensity of protein-like substances for the LB-EPS and TB-EPS extracted from AnMBR at 25 °C were stronger than those extracted from AnMBR at 35 °C. The trends in the EEM spectra intensity for LB-EPS and TB-EPS coincided with the fouling tendencies monitored for the two AnMBRs at 35 and 25 °C. It was demonstrated that the protein-like substances in the LB-EPS and TB-EPS had an important effect on the membrane fouling development in the AnMBR at 35 °C and the AnMBR at 25 °C. Taking into consideration the results of EEM fluorescence spectra analyses, indicating that the EPS in the AnMBR at 25 °C showed more proteinaceous characteristics, it can be inferred that the protein-like substances are abundant in EPS of the AnMBR at 25 °C, which would cause severe membrane fouling.

### 3.5. Morphology Characteristics of the Sludge Flocs and Their Effects on Membrane Fouling

**Figure 5:**
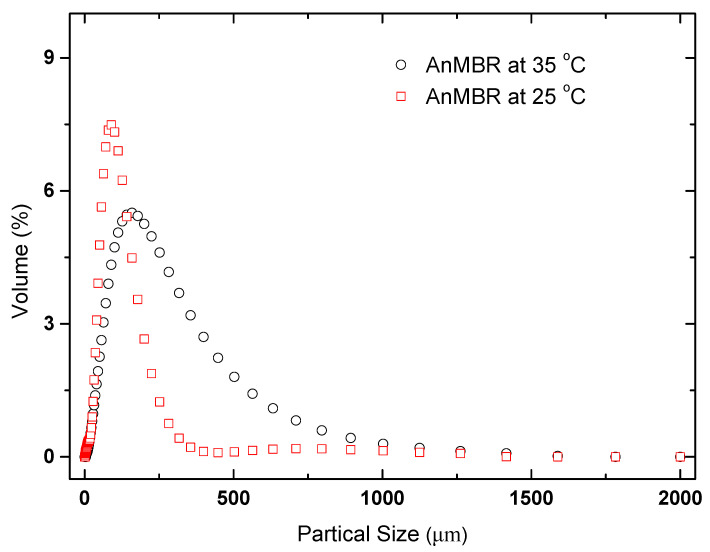
The particle size distribution (PSD) of sludge flocs in the two AnMBRs at 35 and 25 °C.

The PSD of activated sludge in the two AnMBRs at 35 and 25 °C is listed in Figure 5. There are obvious differences between the two AnMBRs at 35 and 25 °C, indicating that operation temperature had significant impact on PSD in the two AnMBRs at 35 and 25 °C. At the steady state of the AnMBR at 35 °C, the particle size of 90% sludge flocs was less than 383.579 μm, and the particle size of 90% sludge flocs was less than 180.043 μm in the AnMBR at 25 °C. A previous study reported that sludge particles with smaller particle sizes could be easily deposited on the membrane surface to form a cake layer [30]. The reason was that the back transport velocity of the particles reduced with the decrease of their size [31]. Thus, one of the reasons for the more severe membrane fouling trend in the AnMBR at 25 °C could be attributed to the smaller particle size of flocs, inducing serious cake formation.

**Figure 6:**
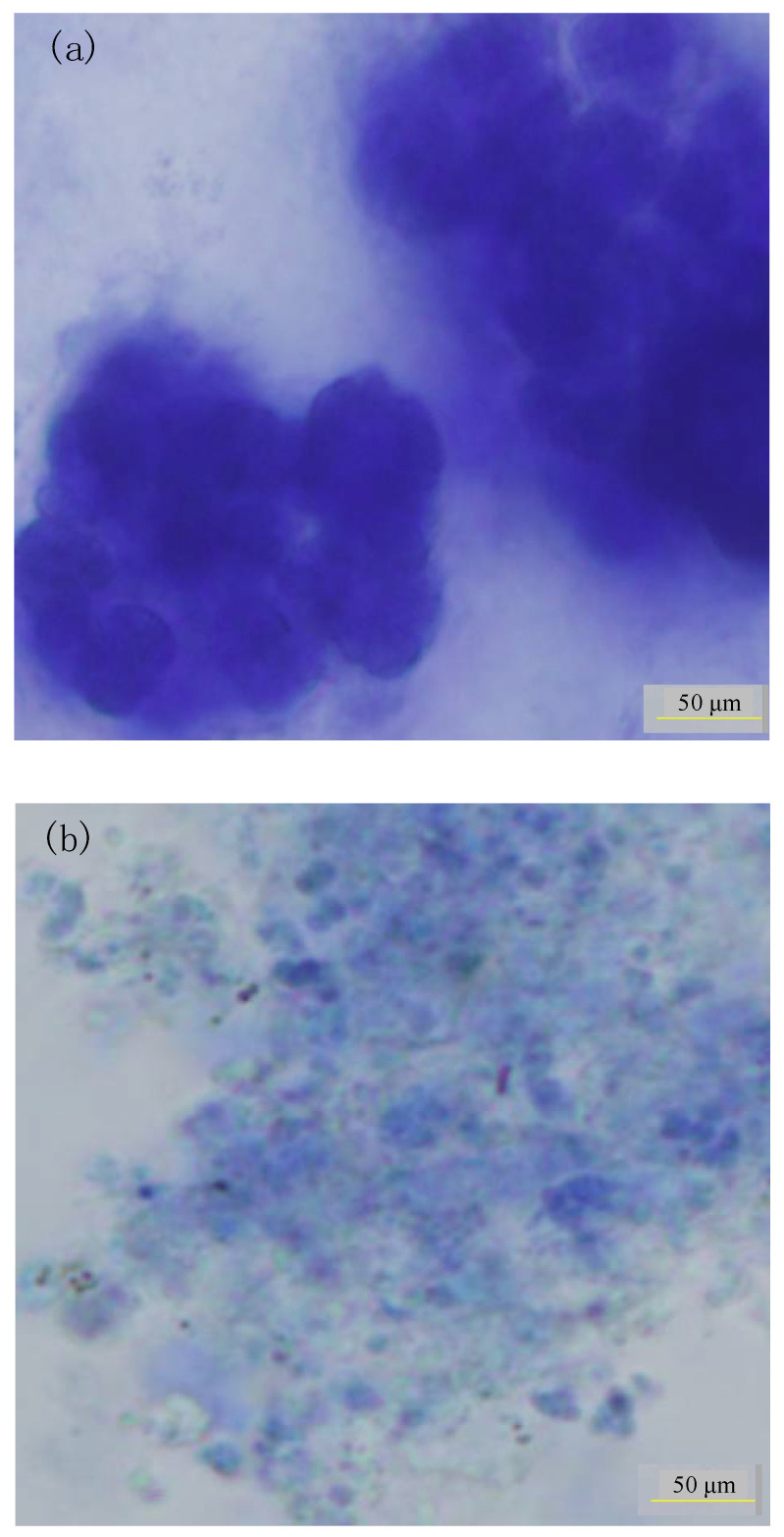
The sludge floc size and structure in the AnMBRs under 35 (**a**) and 25 °C (**b**) conditions.

The sludge floc structure in the two AnMBRs at 35 and 25 °C is shown in Figure 6. It was illustrated that the sludge floc size was lower in the AnMBR with 25 °C condition than that in the AnMBR with 35 °C condition. Compared to that in the AnMBR under 35 °C conditions, the decrease in floc size in the AnMBR under 25 °C conditions could be owing to the effect of the lower temperature on the growth rate of anaerobic microorganisms, which caused the lower sludge aggregation.

As seen from the microscopic analysis, the amount of fine flocs in the AnMBR at 25 °C was more than that in the AnMBR at 35 °C. Consequently, the greater quantity of small sludge flocs in the AnMBR at 25 °C might have contributed to the more severe membrane fouling compared to the AnMBR at 35 °C. Thus, the membrane fouling propensity for the AnMBR at 25 °C was higher than that for the AnMBR at 35 °C. It has been indicated that more LB-EPS in EPS could reduce floc bioflocculation and affect the floc structure [8]. Therefore, the LB-EPS concentration was higher in the AnMBR at 25 °C compared to that in the AnMBR at 35 °C, which may result in the higher fouling propensity.

## 4. Conclusions

In this study, sludge floc characteristics were analyzed and their effect on membrane fouling was researched for anaerobic membrane bioreactors (AnMBR). The temperature differences between the two AnMBRs resulted in a number of different physical and biochemical properties of mixed liquor that seemed to be related to the differences in the fouling behaviors of the two types of sludge. It was found that the mixed liquor in the AnMBR at 25 °C exhibited consistently higher membrane fouling propensity than the mixed liquor in the AnMBR at 35 °C. The content of EPS in the sludge flocs showed a relationship with fouling tendency. Mixed liquor samples with higher polysaccharide and protein content and protein/polysaccharide ratio in EPS were observed to have greater potential to foul membranes during the AnMBR operation at 25 °C. Meanwhile, the EEM spectra peak intensities of protein-like substance for the LB-EPS and TB-EPS extracted from the AnMBR at 35 °C were stronger than those extracted from the AnMBR at 35 °C. The content of EPS (especially polysaccharide and protein substances) in the activated sludge mixed liquor was concluded to be a key index to assess the fouling propensity. Furthermore, the sludge particle size was smaller and a larger amount of fine flocs was found in the AnMBR at 25 °C. Thus, one of the reasons for the raised membrane fouling potential in the AnMBR at 25 °C could be attributed to the EPS and sludge floc characteristics.
